# Parallel Reduction in Skeletal Density During Benthic to Pelagic Transitions in Baikal Sculpins

**DOI:** 10.1093/iob/obag026

**Published:** 2026-06-05

**Authors:** B A Gutierrez, O Larouche, S Loetzerich, M E Gerringer, K M Evans, A Aguilar, S Kirilchik, M W Sandel, J M Daane

**Affiliations:** Department of Biology and Biochemistry, University of Houston, Houston, TX 77204, USA; Department of Biology, Western Carolina University, Cullowhee, NC 28723, USA; Department of Biology and Biochemistry, University of Houston, Houston, TX 77204, USA; Department of Biology, State University of New York at Geneseo, Geneseo, NY 14454, USA; Department of Biosciences, Rice University, Houston, TX 77005, USA; Department of Biological Science, California State University Los Angeles, Los Angeles, CA 90032, USA; Limnological Institute, Russian Academy of Sciences, Irkutsk 664033, Russia; Department of Wildlife, Fisheries and Aquaculture, Mississippi State University, Mississippi State, MS 39762, USA; Forest and Wildlife Research Center, Mississippi State University, Mississippi State, MS 39759, USA; Department of Biology and Biochemistry, University of Houston, Houston, TX 77204, USA

## Abstract

Habitat transitions are a major driver of morphological evolution. Teleost fishes have repeatedly transitioned from benthic to pelagic habitats, often evolving predictable changes in body shape that enhance hydrodynamic efficiency. While freshwater sculpins (Cottidae, Perciformes) are usually benthic, two genera in Lake Baikal, *Comephorus* and *Cottocomephorus*, have independently evolved into mid-water niches. As sculpins lack a swim bladder, these lineages instead improved buoyancy through reduced skeletal density and increased lipid stores. Using micro-computed tomography and two-dimensional morphometrics, we characterized skeletal evolution in six benthic, two benthopelagic, and two pelagic species across the Baikal sculpin radiation. Parallel changes to mineral density, porosity, and thickness evolved independently in pelagic and benthopelagic clades, with mineral density showing the most consistent and significant reductions. Density reductions occurred throughout the skull in pelagic and benthopelagic species. The basibranchials and neurocranium exhibited the lowest overall bone density across all cranial elements. While the jaws maintained the highest absolute density values among the bones we measured, they also showed the greatest proportional reduction in density associated with pelagic habitat use, with a 56.86% decrease in percentage hydroxyapatite and a 21.39% increase in porosity. Morphometric analyses further identified parallel evolution toward an elongate body shape, reduced and posteriorly shifted eyes, and elevated fin insertion in pelagic and benthopelagic taxa. These results demonstrate a repeated skeletal lightening and body shape changes accompanying benthic-to-pelagic transitions. This pattern mirrors other benthic-to-pelagic transitions in teleosts that lack swim bladders, highlighting shared biomechanical and microstructural solutions to life in the open water.

## Introduction

The skeleton serves as a critical scaffold for vertebrate morphology, providing structural support, protection for internal organs, anchorage points for muscles, and a reservoir for key minerals such as calcium and phosphorus (Šromová et al. 2023). Because of these essential functions, skeletal elements could be expected to experience stabilizing selection, leading to structural conservatism across evolutionary time (Sansalone et al. 2022). However, many major evolutionary innovations and ecological transitions in vertebrates have arisen through modifications of skeletal structures, allowing lineages to diversify, adapt to new environments, and evolve novel morphologies (Dial et al. 2015).

Teleost fishes, which account for nearly half of all extant vertebrate species (Nelson et al. 2016), exhibit striking diversity in skeletal form and function. The skeleton typically comprises only 3–5% of body mass in bony fishes, but bone is far denser and heavier than other tissues and generates negative buoyancy (Reynolds and Karlotski 1977). Buoyancy has a major impact on the ability of individuals to station hold, swim horizontally, and stabilize posture (Weihs 1973; Webb 2005; Sato et al. 2013; Gleiss et al. 2017; Di Santo and Goerig 2025). Thus, density has a pervasive influence on aquatic locomotion, driving adaptations in both morphology and behavior (Pelster 2009).

To counteract the weight of dense tissues, most teleosts use inflatable swim bladders to maintain neutral buoyancy (Pelster 2009). However, the swim bladder has been lost independently at least 30 times in teleost evolution (McCune and Carlson 2004), often in benthic species that rest on the substrate or in deep-sea fishes where the high pressures make gas inflation difficult (Scholander and Van Dam 1954; Priede 2018). In these cases, alternative buoyancy-control mechanisms have evolved (Pelster 2009). One such buoyancy-control mechanism is the reduction of skeletal density, a buoyancy adaptation also found in non-teleost vertebrate taxa, including deepwater cetaceans (Gray et al. 2007; Pelster 2009).

Changes in skeletal density are common among fishes and can take multiple forms, including reductions in bone size and thickness, increased porosity, or lowered mineral content (Eastman et al. 2014; Gerringer et al. 2021; Martin et al. 2022). Further, fish bones vary widely in structure, appearing spongy, compact, intermediate between bone and cartilage, or consisting primarily of connective tissue or persistent hyaline cartilage rather than fully ossified tissue (Meunier and Huysseune 1991; Eastman et al. 2014). Changes in structure and overall density are often not uniform across the skeleton, reflecting structure-specific trade-offs between minimizing weight and preserving essential functions such as protection and muscle attachment.

Adaptive changes in skeletal density have been drivers of diversification. The cryonotothenioid, or Antarctic notothenioid (Notothenioidei, Perciformes), radiation in the Southern Ocean exemplifies this pattern. A central axis of cryonotothenioid diversification involved vertical habitat expansion to exploit pelagic prey (Eastman 1993). Notothenioids lack swim bladders, instead achieving buoyancy through reduced skeletal density and increased lipid accumulation (Eastman et al. 2014; Eastman 2024). Notably, initial reductions in skeletal density evolved prior to the adaptive radiation in the Antarctic Southern Ocean, which is thought to have facilitated subsequent diversification into pelagic niches (Daane et al. 2019).

A similar, though less studied, radiation has occurred among the sculpins (Cottidae, Perciformes) in Lake Baikal. At 1642 m maximum depth (De Batist et al. 2006), Lake Baikal is the world’s oldest and deepest freshwater lake. Unique among lake habitats, Lake Baikal is well-oxygenated along the entire water column. This oxygenation supports life in marine-like habitats such as hydrothermal vents, cold seeps, and bathypelagic zones, and has fostered more than 1500 endemic species and multiple animal adaptive radiations (Ravens et al. 2000; Macdonald et al. 2005; Stelbrink et al. 2015; Sandel et al. 2025). Cottid sculpins dominate the fish fauna of Lake Baikal, with 42 described cottid species (38 endemic) comprising 57% of Baikal fish species diversity (Matveyev and Samusenok 2015; Bogdanov 2023; Sandel et al. 2025). Like notothenioids, Baikal sculpin diversification is marked by vertical habitat expansions, particularly colonization of deep benthic habitats, with elevated speciation rates and subsequent morphological diversification in the bathybenthic clade Abyssocottini (Sandel et al. 2025). Note, within the Baikal sculpins, only Abyssocottini fulfills the formal criteria for adaptive radiation, namely monophyly, rapid speciation, and adaptive divergence driven by natural selection (Schluter 2000a; 2000b; Glor 2010; Sandel et al. 2025), with other Baikal sculpin clades lacking increases in speciation rates.

**Fig. 1:**
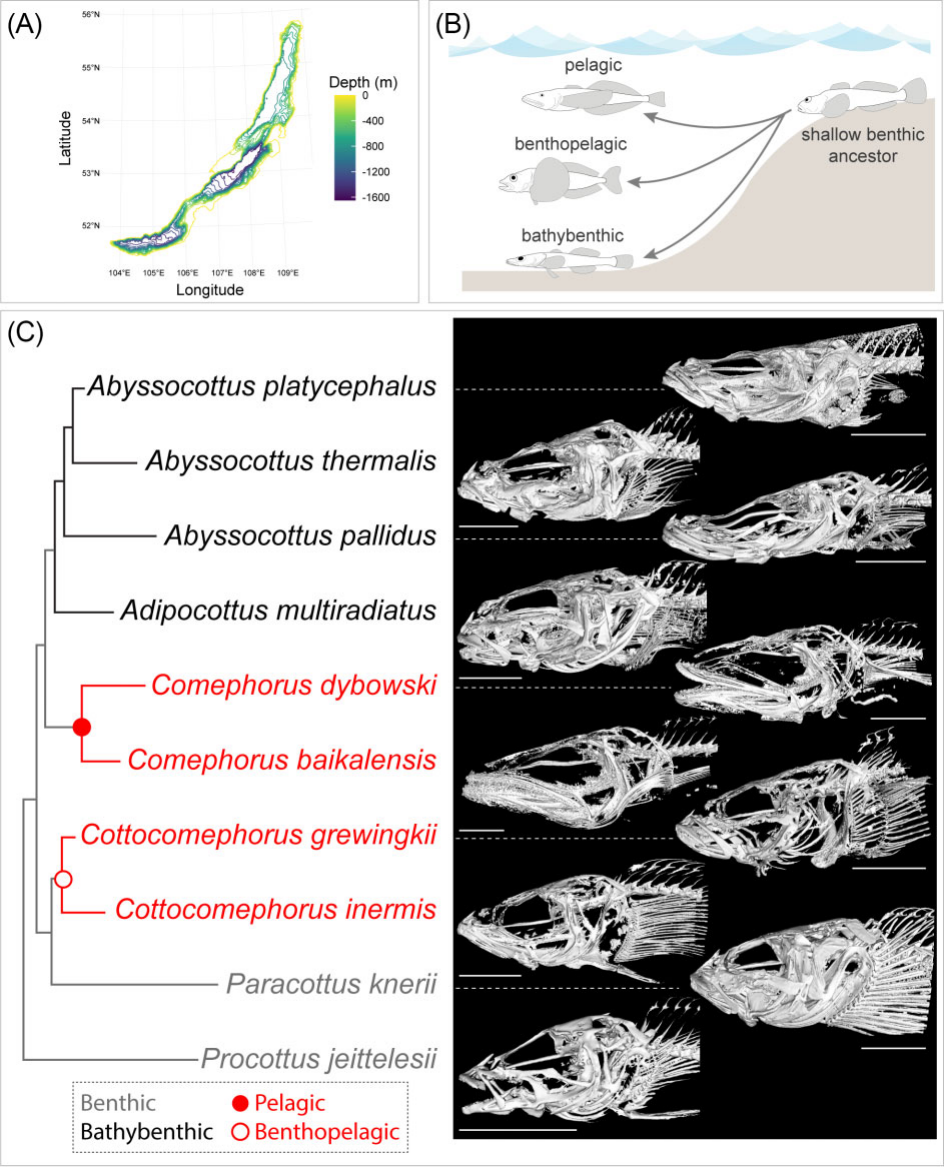
Benthic-to-pelagic transitions during the Baikal sculpin adaptive radiation. (A) Bathymetric map of Lake Baikal [data from (De Batist et al. 2006)]. (B) The adaptive radiation of the Baikal sculpins from a shallow benthic common ancestor into bathybenthic, benthopelagic, and pelagic species. (C) Cladogram and skull reconstructions at optimum brightness for each scan from selected species of Baikal sculpins. Red indicates pelagic (filled circle, *Comephorus*) or benthopelagic (unfilled circle, *Cottocomephorus*) species, gray indicates benthic species and black benthopelagic species. Cladogram and habitat associations adapted from ancestral state reconstructions in (Sandel et al. 2025). Skeleton brightness optimized for each scan. Scale bars in C represent 10 mm.

Sculpins have also diversified into the vast pelagic zone of Lake Baikal, where 80% of the lake’s surface area is at depths exceeding 300 m (Kolokoltzeva 1961; Sideleva 2003) (Fig. 1A). In this habitat, five pelagic sculpin species dominate, comprising ~80% of total fish biomass (Sideleva 2003). The five pelagic sculpins are distributed between two genera, the fully pelagic *Comephorus* (*C. baikalensis, C. dybowski*) and benthopelagic *Cottocomephorus* (*Cot. comephoroides, Cot. grewingkii*, and *Cot. inermis*). These genera represent two independent benthic-to-pelagic transitions (Sandel et al. 2025)*. Comephorus* species are fully pelagic, engage in diel vertical migrations for nocturnal feeding, and have evolved ovoviviparity, which removes a dependency on benthic egg nesting (Taliev 1955; Sideleva 2003). In contrast, *Cottocomephorus* species feed in midwater during the day but remain tied to the benthos for spawning, nesting, and resting (Sideleva 2003). The two genera also differ in their primary pelagic prey items, which is reflected in morphological traits relevant for trophic ecology. As adults, *Comephorus* preys on a combination of large and mobile *Macrohectopus* amphipods and other fish (Anoshko et al. 2002). To facilitate prey capture, *Comephorus* has a large mouth opening with elongated jaws (16% of body length) that are lined on the exterior with needle-like teeth (Aleev 1963; Sideleva 2003). A similar elongation of the jaws has been documented in benthic Baikal sculpins associated with consumption of larger prey items and transitions toward ichthyophagy (Tolmacheva 2010; Tolmacheva and Bun 2019). *Cottocomephorus* largely target zooplankton and *Epischurella* copepods, possessing a mouth half the size of that in *Comephorus*, toothless outer jaws, and relatively long gill rakers (Taliev 1955; Sideleva and Kozlova 1989; Sideleva 2003).

Like notothenioids, pelagic and benthopelagic Baikal sculpins diversified from a negatively buoyant benthic common ancestor lacking a swim bladder. Whereas benthic sculpins typically have skeletal ash weights above 3%, pelagic and benthopelagic forms fall below this threshold, with *C. baikalensis* achieving near-neutral buoyancy with skeletal ash weight as low as 1.6–2.5% and a specific gravity of 1.010 (Taliev 1955; Sideleva 2003). *Comephorus* is reported to have thinner neurocranial and pectoral fin bones and increased bone porosity, though detailed architectural descriptions are scarce and historically limited to these broader, coarse metrics (Sideleva 2003). Low skeletal weight, combined with high levels of corporeal lipids and elongated pectoral fins (Jakubowski et al. 2003; Sideleva 2003), enables *Comephorus* to passively hover in the water column, in contrast to the typically active swimming of pelagic species.

While foundational work by Taliev (1955) established an early framework for how skeletal changes facilitate benthic-to-pelagic transitions in sculpins, modern micro-computed tomography (micro-CT) approaches allow us to evaluate these shifts at a microarchitectural level and across the phylogeny. Here, we characterize skeletal variation across the Baikal sculpin phylogeny to assess trait-habitat associations and underlying modes of density reduction. We test the hypothesis that reductions in mineral density, porosity, and thickness independently evolved in secondarily pelagic sculpins and are explicitly associated with mid-water habitat usage over other ecological parameters, such as depth. This study provides new insights into how skeletal shifts facilitate benthic-to-pelagic transitions, with broader implications for understanding adaptive radiations in aquatic systems.

## Materials and methods

### Species sampling and habitat scoring

Baikal sculpin specimens were collected from Lake Baikal via trawling, snorkeling, SCUBA, dipnets, funnel traps, and seines, fixed in 10% formalin and stored in 70% ethanol as described previously (St. John et al. 2022; Sandel et al. 2025). We analyzed replicates from ten species in Lake Baikal from four out of the five taxonomic tribes spanning the adaptive radiation (Fig. 1C, Table 1) (Bogdanov 2023): Comephorini (*C. baikalensis* [*n* = 3 individuals], *C. dybowski* [n = 3]), Abyssocottini (*Adipocottus multiradiatus* [n = 4]*, Abyssocottus platycephalus* [*n* = 3], *Ab. pallidus* [*n* = 3], *Ab. thermalis* [n = 2]), Cottocomephorini (*Cot. grewingkii* [*n* = 6], *Cot. inermis* [n = 4]), and Cottini (*Procottus jeittelesii* [*n* = 4]*, Paracottus knerii* [*n* = 5]). Most sampled individuals are within about 70–80% of reported maximum fish total lengths and are within estimated ranges for length at maturity (Table S1) (Koryakov 1964; Jakubowski et al. 2003; Sideleva 2003; Bogdanov 2023). The exceptions are samples of *Pr. jeittelesii*, which are likely juvenile or young adults.

**Table 1:** Cottid species used in this study

Tribe	Species	Common name	*n*	Habitat	Max depth (m)^†^
Abyssocottini	*Abyssocottus platycephalus* (Taliev 1955)	Flathead sculpin	3	Bathybenthic	800
Abyssocottini	*Abyssocottus pallidus* (Taliev 1948)	Slender sculpin	3	Bathybenthic	1200
Abyssocottini	*Abyssocottus thermalis* (Sideleva 2002)	Thermal sculpin	2	Bathybenthic	480
Abyssocottini	*Adipocottus multiradiatus* (Berg 1907)	Spotty-fins sculpin	4	Bathybenthic	900
Comephorini	*Comephorus dybowski* (Korotneff 1905)	Little golomyanka	3	Pelagic	1600
Comephorini	*Comephorus baikalensis* (Pallas 1776)	Big golomyanka	3	Pelagic	1600
Cottocomephorini	*Cottocomephorus grewingkii* (Dybowski 1874)	Yellowfin Sculpin	6	Benthopelagic	450
Cottocomephorini	*Cottocomephorus inermis* (Jakowlew 1890)	Big-eyed longfin sculpin	4	Benthopelagic	500
Cottini	*Paracottus knerii* (Dybowski 1874)	Stone sculpin	5	Benthic	200
Cottini	*Procottus jeittelesii* (Dybowski 1874)	Jeitteles’s sculpin	4	Benthic	200

† Maximum depth estimates aggregated from Sandel et al. (2025) and Sideleva (2003).

* Note, many species are eurybathic and have a wide vertical distribution.

Species in our dataset were classified as either benthic (*n* = 6 species) or pelagic (*n* = 4) (Table 1, Fig. 1B, C) (Sideleva 2003). Within the pelagic group, species of the genus *Comephorus* (*n* = 2) represent a true transition to a pelagic lifestyle, while species of the genus *Cottocomephorus* are considered benthopelagic (*n* = 2), occupying both benthic (bottom-dwelling) and pelagic (open water) zones (Sideleva 2003).

### Scanning of specimens

We performed micro-CT scans of selected Baikal sculpin species using a Bruker SkyScan 1273 micro-CT scanner configured with 70 kV X-ray voltage, 214 µA current, medium focal spot size, and 0.3° rotation step. Scan lengths ranged from 145 to 245 mm with voxel sizes between 12 and 20 µm, optimized for each specimen’s size. Two calcium hydroxyapatite phantom markers (25% and 75% density) were included in each scan to calibrate bone density measurements to scan brightness. Micro-CT data were reconstructed using Bruker NRecon v.2.1.0.1, cropping the region from snout to the sixth or seventh vertebra. Beam hardening correction (44%) minimized polychromatic X-ray artifacts, with dynamic image range set between 0.00 and 0.095 for optimal contrast. All image processing and segmentation used NRecon, DataViewer, and Amira Version 6.0.1 on Windows OS, adapting methodologies from Martin et al. (2022) and Buser et al. (2020) for compatibility with current Bruker software. For additional details on scanning and image processing, see Supplementary Methods.

### Bone mineral density, bone volume fraction, and bone Thickness

**Fig. 2:**
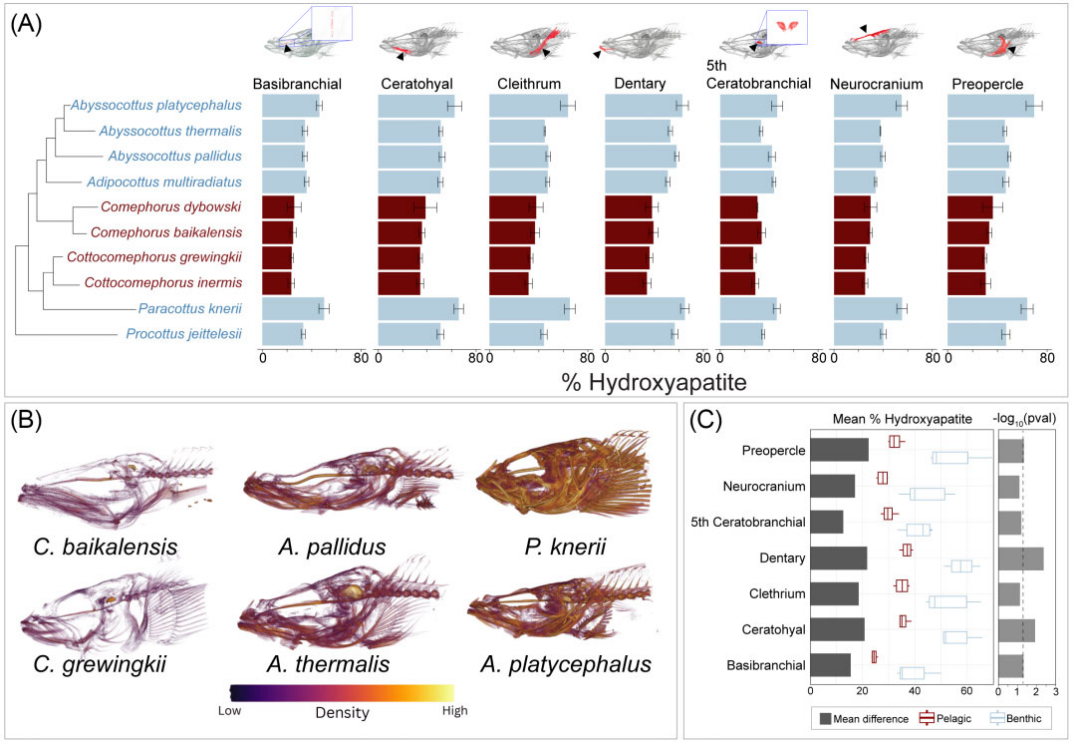
Patterns of skull bone mineral density across the Baikal sculpin radiation. (A) Mean percentage of hydroxyapatite, comparing benthic and pelagic/benthopelagic (Comephorus and Cottocomephorus) groups. (B) Skull density heatmaps of representative Baikal sculpins, including two pelagic species (*C. baikalensis* and *Cot. grewingkii*) and four benthic species (*Ab. pallidus, Ab. thermalis, Ab. platycephalus*, and *P. knerii*). (C) Mean differences in hydroxyapatite percentage between benthic and pelagic groups, with ANOVA *P*-values transformed via −log₁₀ for visualization (Table S3). Dashed line indicates a *P*-value of 0.05.

We estimated three metrics of skeletal density and morphology: bone mineral density, porosity, and thickness. We focused on cranial elements critical for feeding and structural integrity, including the preopercle, ceratohyal, fifth ceratobranchial, dentary, dorsal neurocranium, basibranchials, and cleithrum (see Fig. 2A) (Gregory 1959). The dorsal neurocranium makes up the rigid roof of the skull that protects the brain. The dentary is the main, tooth-bearing bone of the lower jaw. The preopercle supports the posterior of the cheek just ahead of the gill cover. The cleithrum is a major bone of the pectoral girdle that connects the pectoral fins to the back of the skull through intervening elements. The ceratohyal, a key component of the hyoid arch, forms part of the mouth floor and connects ventrally to the basibranchials, a series of midline bones that support and link the gill arches. Finally, the fifth ceratobranchial is a modified, often tooth-bearing bone that serves as a pharyngeal jaw for processing food.

Bone mineral density was assessed using the hydroxyapatite phantoms as reference standards, converting grayscale intensity values from the scan data into mineral density measurements through relative pixel brightness comparisons, which translates to a percentage of hydroxyapatite value (% HA). For each scan, we determined mean pixel brightness values from these markers in full and developed scan-specific linear models using mean pixel brightness as the predictor and known % HA as the response variable. These models allowed us to convert mean pixel brightness measurements from segmented bones into predicted % HA values, providing standardized mineral density assessments across skeletal elements (Martin et al. 2022).

Bone volume fraction (BVF) offers valuable insight into bone density by quantifying the spatial organization of bone tissue. We used this metric to assess and compare bone porosity across Baikal sculpins. Using 3D Slicer (Version 5.6.2) (Kikinis et al. 2014), we obtained the two necessary measurements for BVF calculations: bone volume (BV) and total volume (TV). BV represents the actual segmented BV, where higher porosity results in lower BV values. TV measures the same region with all voids filled, representing a hypothetical solid bone structure (Fig. S1). The BVF is derived from the BV/TV ratio, ranging from near-zero (highly porous) to one (completely solid). We computed BVF for four of our seven target bones, excluding the cleithrum and preopercle due to issues in void filling accuracy for arched bones and the basibranchial due to its small size and low mineral density.

We conducted comparative thickness analyses of the same four bones examined in our BVF assessment across Baikal sculpin species. For each segmented bone, thickness values were computed in 3D Slicer and stored as a scalar field on the surface model, with one thickness value assigned to each mesh vertex. Using a custom Python script, we extracted these per-vertex thickness scalars and calculated the mean thickness across all surface points for each bone. These values represent average geometric bone thickness (in millimeters) derived directly from the model geometry rather than material density. The resulting mean thickness values provide a standardized, specimen-level descriptor of bone geometry suitable for cross-taxon comparisons and correlation with other structural metrics such as BVF.

Bone mineral density was computed using Amira (Version 2020.3) on Windows OS. We used built-in segmentation, thresholding, and material statistics tools to help calculate mean pixel brightness and use that metric to translate it to our % HA measurements. Our BVF and thickness metrics were computed using 3D Slicer on Windows OS. We utilized built-in modules, including Volume Rendering, Models, Data, Segment Editor, Segment Statistics, Simple Filters, and Probe Volume With Model. For enhanced segmentation and analysis capabilities, we additionally installed the Slicer Morph and Surface Wrap Solidify modules. For further details on all density calculations, see Supplementary Methods.

### Geometric morphometrics analysis

We used two-dimensional geometric morphometrics to examine overall body shape differences between benthic and pelagic sculpins and to investigate the potential relationship between body size and bone properties.

All specimens were photographed in lateral view following a standardized protocol. We positioned each specimen on a dissecting tray using insect pins to stabilize them and mark anatomical landmarks that may be difficult to visualize in photographs (e.g., the base of median fins that often could not be fanned without risking damage to the preserved specimens). Each photograph included a ruler for scale calibration. Images were captured using an Olympus OM D E M1 Mark II camera equipped with an OM System M.Zuiko Digital ED 25mm F1.8 II lens mounted on a copy stand. To maintain optimal image quality across different sized specimens, we adjusted the camera height to 37.5 cm for small specimens, 43.2 cm for mid-sized specimens, and 48.3 cm for large specimens, ensuring consistent focus and framing throughout our dataset.

We digitized morphological features using tpsDig2 (v 2.32) (Rohlf 2017), using 15 fixed landmarks and 53 sliding semilandmarks to comprehensively capture body shape characteristics and fin positioning across all specimens (Fig. S2, Table S2). To standardize our shape data, we performed a generalized Procrustes analysis that accounted for variations in specimen size, position, and orientation, resulting in aligned shape coordinates and centroid size measurements for each individual. Subsequently, we computed species-level average shapes using the mshape function in the geomorph package (Adams and Otárola-Castillo 2013).

The following replicates were used per species for geometric morphometrics: *C. baikalensis* (*n* = 5 individuals), *C. dybowski* (*n* = 5), *Ad. multiradiatus* (*n* = 7)*, As. platycephalus* (*n* = 9), *Ab. pallidus* (*n* = 6), *Ab. thermalis* (*n* = 2), *Cot. grewingkii* (*n* = 8), *Cot. inermis* (*n* = 4), *Pr. jeittelesii* (*n* = 4), and *Pa. knerii* (n = 7).

### Comparative methods

We conducted all comparative analyses using a recent phylogeny (Sandel et al. 2025), which we pruned to include only the species present in each dataset. To examine habitat effects on bone metrics, we performed one-way phylogenetic Analysis of Variance (ANOVA) using the aov.phylo function from the geiger package (v 2.0.11) in R (Pennell et al. 2014). We assessed statistical significance by comparing observed values against a null distribution generated from 1000 simulations under a Brownian motion model. Given that significance testing uses a simulation approach, we performed each ANOVA 1000 times and reported the mean of the distribution of *P*-values. These analyses compared average bone densities between benthic and pelagic Baikal sculpin species. Prior to ANOVA, normality of each response variable was assessed using Shapiro–Wilk tests conducted separately for each bone, consistent with the bone-specific ANOVA design. All measurements showed no significant departure from normality (*P* > 0.05) except for neurocranium porosity (*P* = 0.04505). We applied identical approaches to evaluate habitat effects on average bone porosity and thickness, creating separate ANOVA models for each bone of interest.

To quantify the magnitude of habitat-associated differences, we calculated standardized effect sizes using Hedges’ *g* for each bone metric (density, porosity, and thickness) comparing benthic and pelagic species. Hedges’ *g* was selected because it provides an unbiased estimate of standardized mean differences under small sample sizes, which is appropriate for our limited number of species per habitat category. Effect sizes were computed as the difference in habitat means divided by the pooled standard deviation, with a small-sample correction factor applied following Hedges and Olkin (1985). Positive values indicate higher trait values in pelagic taxa, whereas negative values indicate higher values in benthic taxa. We interpret these effect sizes alongside phylogenetic ANOVA results to assess whether biologically meaningful habitat differences may exist even when statistical power is limited. Following conventional benchmarks, absolute values of approximately 0.2, 0.5, and 0.8 were considered small, moderate, and large effects, respectively, while emphasizing effect size magnitudes and confidence intervals over categorical thresholds.

To investigate relationships between bone properties, we conducted phylogenetic multiple linear regressions using the phylolm function from the phylolm R package (Ho and Ané 2014). These analyses tested for linear relationships between bone density (response variable) and both porosity and thickness (predictor variables), incorporating data from all four bones measured for porosity and thickness. This allowed us to assess whether bone perforation patterns and thickness influence mineral density.

We also examined potential correlations between density metrics among bones, between density metrics and body size, and between density metrics and habitat depth using Spearman correlation coefficients. For a measure of body size, we used the species averages of centroid size values derived from their 2D geometric morphometrics analysis. Prior to analysis, all quantitative variables were transformed into phylogenetically independent contrasts using the pic function from the ape R package (Paradis et al. 2004).

We analyzed shape variation patterns in Baikal sculpins using two complementary approaches. First, we conducted a principal components analysis of shape coordinates using the gm.prcomp function in geomorph to identify major axes of morphological variation. Second, we performed phylogenetic Procrustes ANOVAs with the procD.pgls function (geomorph package) to test for habitat effects on body shape, assessing significance against a null distribution generated from 1000 permutations of tip data. The Principal Component Analysis (PCA) results revealed that PC1 effectively distinguished benthic and pelagic species, prompting additional phylogenetic ANOVAs in geiger using species scores along this first principal component to further quantify habitat related shape differences.

## Results

### Independent reductions in skeletal density across pelagic sculpins

Skeletal density and structure were quantified from 3D skeletal reconstructions using three primary metrics: mineral density, porosity, and bone thickness. Notably, thickness is a measure of skeletal morphology rather than a density metric. Mineral density varied substantially across the dataset, both among individual bones and between species. The basibranchials and neurocranium consistently had the lowest densities, while the dentary exhibited the highest mineral density values (Fig. 2). There were distinct species-specific differences in overall mineral density that were consistent across all measured bones. *Pa. knerii* and *Ab. platycephalus* showed the highest mineral density, while *Ab. pallidus* and *Ab. thermalis* had intermediate levels (Fig. 2A, B). The pelagic species in the dataset (*Cot. grewingkii, Cot. inermis, C. dybowski*, and *C. baikalensis*) exhibited the lowest mineral densities for each bone (Fig. 2A, C). Although mineral density was universally reduced in pelagic sculpins compared to benthic taxa, certain elements showed disproportionately larger decreases compared to benthic cottids, particularly the preopercle, dentary, and ceratohyal (Fig. 2C). These reductions in mineral density evolved in parallel in *Comephorus* and *Cottocomephorus* (Fig. 2A).

**Fig. 3:**
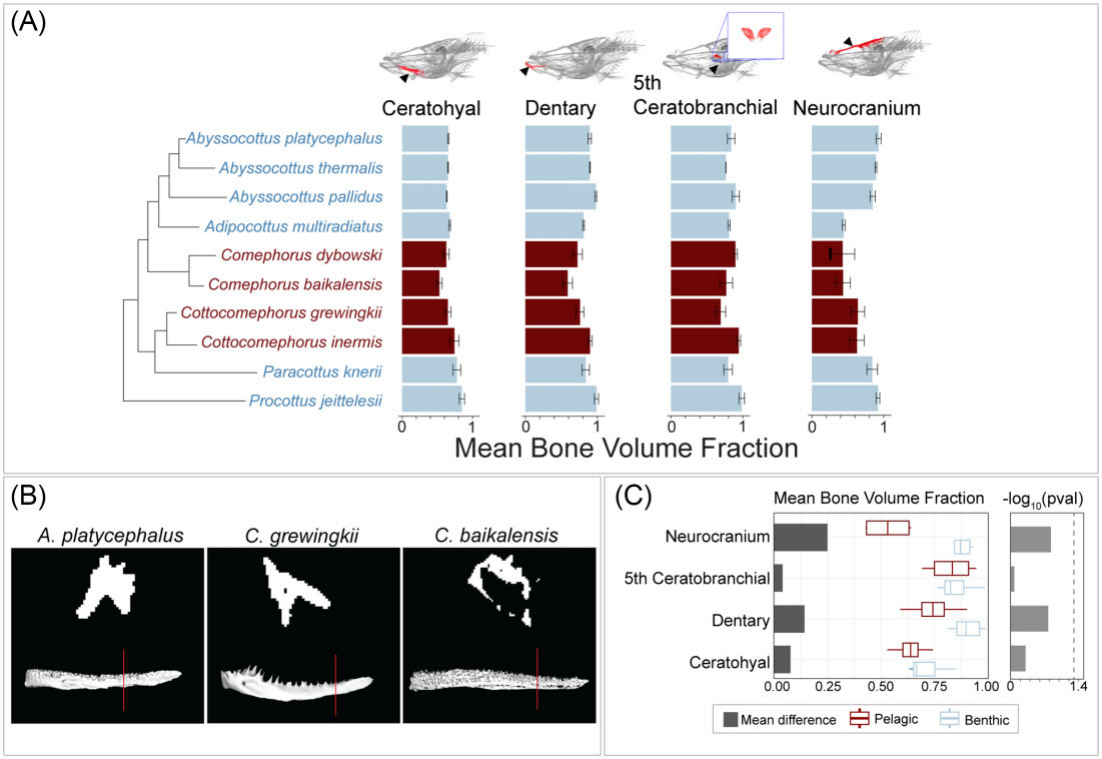
Patterns of bone porosity across the Baikal sculpin radiation. (A) Mean BVF, a measure of porosity, in selected Baikal sculpin species, comparing benthic and pelagic/benthopelagic (Comephorus and Cottocomephorus) groups. (B) Dentary cross-sections (taken at the plane indicated by lines) highlighting differences in porosity between representative species. (C) Mean BVF differences between benthic and pelagic groups, with ANOVA *P*-values transformed via −log₁₀ for visualization (Table S3). Dashed line indicates a *P*-value of 0.05.

We estimated bone porosity using BVF, defined as the proportion of a segmented skeletal element’s volume that is occupied by mineralized tissue. The dentary, ceratohyal, and fifth ceratobranchial maintained relatively consistent porosity levels with minimal variation between species (Fig. 3). The neurocranium displayed the greatest variation in porosity, with increases in the pelagic sculpins (*C. baikalensis, C. dybowski, Cot. grewingkii*, and *Cot. inermis*) as well as the benthic *Ad. multiradiatus* (Fig. 3A). The dentary also showed distinct porosity differences, though not clearly delineating between benthic or pelagic habitats (Fig. 3A, C). As examples of habitat-specific patterns observed across the various bones, benthic *Ab. pallidus* had among the lowest porosity, benthopelagic *Cot. grewingkii* showed an intermediate porosity that is common among other benthic sculpins, while *C. baikalensis*, the only sculpin near neutral buoyancy (Taliev 1955; Sideleva 2003), exhibited the highest porosity, which was consistent across multiple bones (ceratohyal, dentary, neurocranium; Fig. 3A, B).

**Fig. 4:**
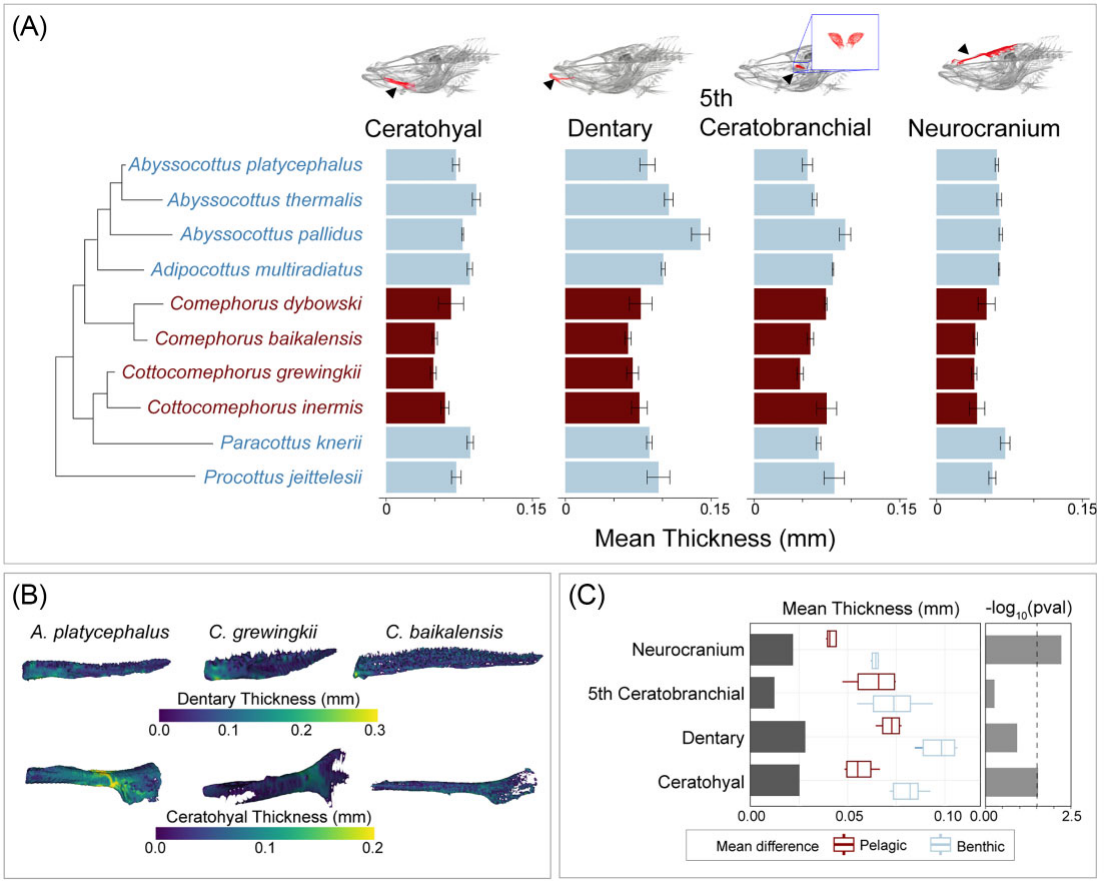
Variation in bone thickness across the Baikal sculpin phylogeny. (A) Mean bone thickness in selected Baikal sculpin species, comparing benthic and pelagic/benthopelagic (Comephorus and Cottocomephorus) groups. (B) Three-dimensional models of dentary and ceratohyal bones from representative specimens overlaid with a heatmap of bone thickness. (C) Mean thickness differences between benthic and pelagic groups, with ANOVA *P*-values transformed via −log₁₀ (Table S3). Dashed line indicates a *P*-value of 0.05.

Bone thickness also varied across the Baikal radiation (Fig. 4). The neurocranium had the lowest average thickness, whereas the dentary had the greatest thickness (Fig. 4A). The bathybenthic species *Ab. pallidus, Ab. thermalis*, and *Ab. platycephalus* consistently had the thickest bones. With the exception of the fifth ceratobranchial, *Cottocomephorus* and *Comephorus* had the thinnest ceratohyal, dentary, and neurocranium. As with mineral density, reductions in thickness evolved in parallel in both pelagic clades and are reflected across multiple bony elements.

To test if the apparent density changes correlate with the benthic–pelagic transition, we used phylogenetic ANOVAs to examine how each bone metric varies across the habitat axis (Table S3). Despite the low statistical power of a phylogenetic ANOVA given the limited number of species included in this analysis, water column position had significant effects on mineral density in the ceratohyal (*P* = 0.014, *g* = −3.27), and dentary (*P* = 0.0047, *g* = 4.19), and marginal effects in the preopercle (*P* = 0.052, *g* = 2.31), basibranchial (*P* = 0.054, *g* = 2.29), cleithrum (*P* = 0.085, *g* = −1.99), fifth ceratobranchial (*P* = 0.071, *g* = −1.08), and neurocranium (*P* = 0.090, *g* = −1.95) (Fig. 2C, Table S3). Notably, the ceratohyal (*g* = −3.27), basibranchial (*g* = −2.29), and preopercle (*g* = −2.31) exhibited particularly strong effect sizes, consistent with pronounced habitat-associated divergence.

For bone thickness (Fig. 4C, Table S3), significant relationships with habitat were found for the neurocranium (*P* = 0.0069, *g* = −3.84) and ceratohyal (*P* = 0.034, *g* = −2.62), while no significant associations were detected for the dentary (*P* = 0.13, *g* = −1.69) or fifth ceratobranchial (*P* = 0.57, *g* = −0.64). Despite this mixed statistical support with our low sample sizes, effect sizes again indicated substantial differences between habitat groups, particularly for the neurocranium (*g* = −3.84) and ceratohyal (*g* = −2.62), suggesting strong reductions in thickness in pelagic species.

In contrast, bone porosity showed no statistically significant relationship with ecological position for any cranial element (Fig. 3C, Table S3; all *P* > 0.16). Correspondingly, effect sizes for porosity were generally moderate (−0.72 > *g* > −1.56), indicating some differences between benthic and pelagic taxa, but weaker and less consistent than those observed for mineral density and thickness.

### Correlations between bones and morphological variables

We next tested whether variation in density metrics was correlated across bones. To do this, we performed phylogenetic regressions using thickness, and porosity as predictors and mineral density as the response variable. These models showed very weak relationships between the three skeletal metrics, with adjusted *R*² values below 0.5 for all bones. Porosity showed a marginal association with mineral density only in the neurocranium (*P* = 0.0559), but model fit was still low (*R*² = 0.44), and thickness was not a significant predictor of mineral density for any bone examined. Separate regression models run for each predictor confirmed this result. Overall, there is little evidence that changes in mineral density, porosity, and thickness are tightly coordinated across cranial bones.

Because body size can influence bone density in fishes (Hamilton et al. 1981; St. John et al. 2022), we also examined correlations between centroid size and each skeletal metric. Bone mineral density and porosity were both negatively correlated with size, while for thickness, Spearman rank correlations were either very weak or slightly positive (Table S4). The negative correlation signal is likely influenced by *Comephorus*, which includes some of the largest specimens in the dataset. Overall, the relationship between body size and cranial skeletal properties appears limited, and no bone showed strong evidence that larger individuals have systematically denser, less porous, or thicker cranial elements.

Finally, we examined correlations in density metrics across distinct skeletal elements. Bone mineral density was highly positively correlated among most bones (Spearman’s ρ ≥ 0.88), with the notable exception of the fifth ceratobranchial, which showed weaker, though still positive, correlations with other bones (ρ = 0.42–0.7; Fig. S3). Thickness and porosity were less consistently correlated across bones, with the strongest relationships observed between the ceratohyal and neurocranium (ρ = 0.57 for thickness, ρ = 0.60 for porosity; Fig. S3). The fifth ceratobranchial had weak negative correlations with ceratohyal thickness (ρ = −0.22) and neurocranium porosity (ρ = −0.18; Fig. S3). Overall, while in general density patterns are broadly correlated across bones, specific elements show distinct variation.

### Parallel shifts in body shape during benthic-to-pelagic transitions

**Fig. 5:**
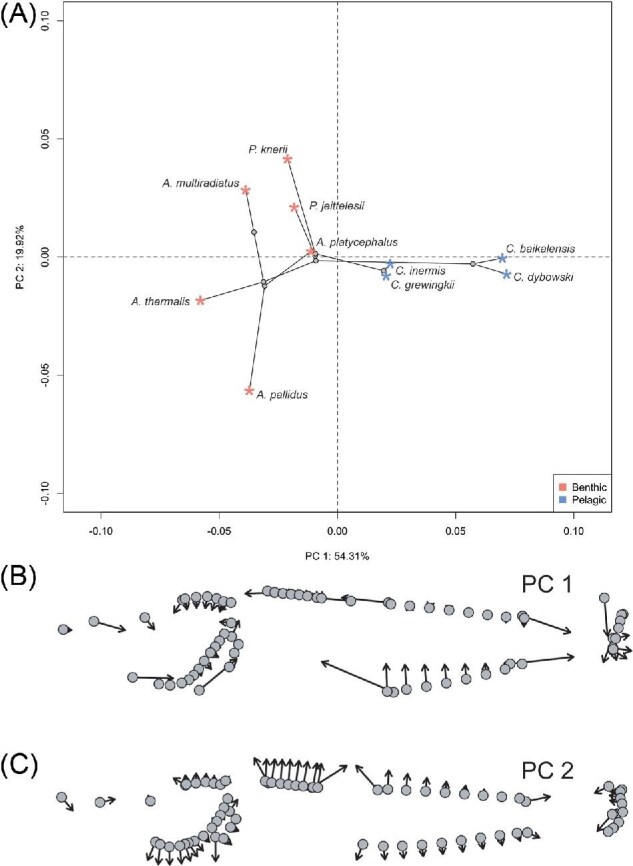
Parallel modifications to body shape during the benthic to pelagic transition in Lake Baikal sculpins. (A) Phylomorphospace (i.e., a projection of the phylogenetic relationships in the morphospace) showing the first two axes of a principal components analysis of the shape data. (B–C) Plot of vector displacements illustrating the changes in shape between the two extremes along PC1 (B) and PC2 (C). Gray dots represent the extreme shape toward the negative end of the axis, while the tips of the vectors represent the extreme shape toward the positive end.

In addition to skeletal density, benthic-to-pelagic transitions typically involve coordinated changes to overall body shape, trending toward a slender, elongate body shape, furcate caudal fins, and a narrow caudal peduncle (Friedman et al. 2020a; Rincon-Sandoval et al. 2020). Principal component analysis of shape data revealed distinct morphological patterns that are consistent with a recent analysis of Baikal sculpin shape variation (Sandel et al. 2025) (Fig. 5A). The first principal component (PC1) (54.31% of variation) clearly separated benthic and pelagic/benthopelagic sculpins, while the second axis (19.92% of variation) primarily reflected differences among benthic species. Pelagic and benthopelagic sculpins showed consistent morphological differences along PC1 relative to the benthic species, including smaller, posteriorly positioned eyes, wider fin bases, closer dorsal fin spacing, higher pectoral fin insertion, and longer, tapering tails (Fig. 5B). Although a multivariate phylogenetic ANOVA found no significant habitat effect on overall shape (*F*_(1,8)_ = 0.92, *P* = 0.44, *Z* = 0.21), a phylogenetic ANOVA focused on the PC1 scores revealed significant divergence between benthic and pelagic groups (*F*_(1,8)_ = 29.08, *P* = 0.02, *g* = −3.14).

Low skeletal density is common in deep-adapted fishes, though it does not correlate linearly with depth (Denton and Marshall 1958; Childress and Nygaard 1973; Cohen 1974; Gerringer et al. 2021). We assessed whether there is an association between skeletal density and depth in Lake Baikal. We used maximum depth values reported for the species in our dataset (Table S1) (Sideleva 2003; Sandel et al. 2025). Importantly, many species are eurybathic and inhabit wide depth ranges, including *Comephorus*, which undergoes diel vertical migrations and can be found from surface to full lake depth (Sideleva 2003). We also examined correlations between bone density metrics and depth through Spearman correlation coefficients. In general there were low and statistically insignificant correlations between depth and mineral density or thickness across all bones (*P* > 0.13, abs(ρ) < 0.53) (Table S5). However, there was a significant negative correlation between depth and ceratohyal porosity (*P* = 0.042, ρ = −0.68) (Table S5). Together, these results indicate that skeletal density metrics are not significantly associated with depth in this system, despite the inclusion of the pelagic and low density *Comephorus* as the deepest fish in the dataset.

## Discussion

### Density patterns across the skull and phylogeny

Parallel reductions in skeletal density evolved in *Cottocomephorus* and *Comephorus* (Figs. 2–4), two genera that independently transitioned from benthic to mid-water habitats (Sandel et al. 2025). The most conspicuous skeletal change in pelagic and benthopelagic species was a skull-wide decrease in mineral density. However, a phylogenetic ANOVA revealed a significant association between mineral density and mid-water habitat usage only in the dentary, ceratohyal. Results in other bones are likely limited by small sample sizes, as our pelagic dataset includes only four species (of the five described pelagic taxa) and captures just two evolutionary transitions (Fig. 2). Detecting significance in these elements, despite small sample sizes, implies that the divergence between mid-water and benthic phenotypes is substantial. Pelagic species also show changes in bone porosity and thickness, but these shifts are weaker and less consistent than those observed for mineral density. *Comephorus baikalensis* had the lowest density across nearly every measurement, corroborating, through different methods, reports from Taliev (1955) and Sideleva (2003) of exceptionally low ash weight and neutral buoyancy.

Density reductions affect skeletal elements differently, reflecting the unique functional demands of each bone. Changes to mineral density were highly correlated across all bones in the skull, though thickness and porosity were more bone-dependent (Fig. S3). The basibranchials and neurocranium had the lowest density across metrics, with particularly low mineral density (Fig. 2), high porosity (Fig. 3), and reduced thickness (Fig. 4). Yet, aside from thickness, these bones did not exhibit the strongest habitat-specific density reductions (Figs. 2–4). In contrast, the dentary showed the largest mineral-density decrease between benthic and pelagic groups. Although associations between these density metrics and benthic and mid-water habitats are not statistically significant after phylogenetic correction, there was consistently high porosity and low thickness within the pelagic jaws, especially the neutrally buoyant *C. baikalensis* (Figs. 2–4). Notwithstanding these reductions to density in pelagic species, the dentary was still denser than the other analyzed bones, a pattern also seen in low-density fishes such as snailfishes (Liparidae) and rattails (Macrouridae) (Gerringer et al. 2021; Martin et al. 2022). This combination of higher density feeding structures and a lower density neurocranium may reflect physical constraints on bone function: the dentary contends with high mechanical loading during feeding, while the neurocranium primarily functions to protect the brain. Supporting this, histological analyses demonstrate that the internal structure of maxillary bones in coastal Baikal sculpins transforms in regions of maximum stress, such as the coronoid process of the dentary, developing compacted tissue and fewer, larger cavities to withstand mechanical loads during feeding (Tolmacheva and Bun 2019). Intriguingly, the primary predators of *Comephorus* are freshwater seals (*Pusa sibirica*), which lack typical molars used to crush prey and swallow these fishes whole (Sideleva 2003; Yakupova et al. 2023). This predator–prey dynamic could influence selection pressures for either trait: relaxed selection for dense protective cranial bones in *Comephorus* and/or on molars in seals without hard-bodied prey. As mechanical load directly impacts fish bone density (Witten and Hall 2015), the element-by-element variation across Baikal sculpins likely reflects a balance between genetic pathways favoring reduced density and plastic responses to mechanical load.

### Bone density: the depth axis

A defining ecological feature of Lake Baikal is its extreme depth, with basins exceeding 1600 m (De Batist et al. 2006). The swim bladder, the primary buoyancy organ in most fishes, becomes increasingly ineffective with depth, as hydrostatic pressure both impedes inflation and compresses gas to high densities (Marshall 1960; Priede et al. 2024). Although some deepwater marine species with swim bladders occur even at hadal depths, such as the cusk eel *Bassogigas profundissimus* (7160 m) and the rattail *Coryphaenoides yaquinae* (7259 m) (Nielsen and Munk 1964; Priede et al. 2024), many deep-sea fishes lack one. Instead, several lineages rely on low-density lipids to maintain buoyancy, as in deep-sea bristlemouths and diel-migrating lanternfishes (Blaxter et al. 1971; Neighbors and Nafpaktitis 1982; Crabtree 1995; Godø et al. 2009; Priede 2017). Additional weight-reducing modifications occur in soft tissues: some species possess low-density “watery” muscles (Crabtree 1995; Tuponogov et al. 2008; Priede 2017), gelatinous subdermal matrices with moderately positive buoyancy (Gerringer et al. 2017), or enlarged, fluid-filled crania (Horn et al. 1978).

Bone, one of the densest tissues, often shows reduced density across deep-sea fishes, leading to the hypothesis that skeletal density decreases with depth in fishes (Denton and Marshall 1958; Childress and Nygaard 1973; Cohen 1974; Gerringer et al. 2021). Baikal sculpins show no apparent association between depth and bone density, as the bathybenthic clade has densities similar to shallow benthic species (Figs. 2–4). This pattern mirrors that of rattails, which also show no depth-density correlation, though rattails do possess swim bladders (Martin et al. 2022). Instead, bone density in sculpins is correlated with benthic and mid-water habitat usage, which was also observed in snailfishes; pelagic snailfishes display lower skeletal density, fewer vertebrae, and loss of bony elements such as suction disks (Gerringer et al. 2021). Thus, correlations between skeletal density and depth appear to reflect mid-water habitat usage and the presence or absence of a swim bladder rather than an inherent product of depth adaptation.

### Contrasts to the cryonotothenioid adaptive radiation

Skeletal reduction patterns in Baikal sculpins closely parallel those in Antarctic notothenioids, though the changes to skeletal density within the two clades differ in evolutionary timing and mode. Skeletal density was initially reduced prior to the cryonotothenioid adaptive radiation, with the skeleton becoming even less dense in secondarily pelagic groups (Eastman et al. 2014; Daane et al. 2019). In contrast, Baikal sculpins appear to have evolved reduced density independently within the secondarily pelagic lineages without being preceded by changes to density in benthic relatives (Figs. 2–4).

Our dataset does not allow direct verification of cartilage retention, but the low densities we observed are consistent with highly cartilaginous skeletons, as in notothenioids (Eastman 2024). Interestingly, comparisons of benthic notothenioids to benthopelagic icefishes (Channichthyidae) revealed no consistent differences in mineral density or porosity among bones despite variation in overall density, leading the authors to suggest that bone shape and changes in developmental timing, known as heterochrony, may contribute more strongly to density reduction than bone mineral density and microstructure (Ashique et al. 2022). Many benthic sculpins and notothenioids are pelagic as larvae or juveniles (Buser et al. 2019; Eastman 2024). Retention of juvenile characters into adulthood could facilitate mid-water adaptation in these clades (Taliev 1955; Eastman et al. 2014; Eastman 2024). A similar pattern is also observed in pelagic snailfishes, which retain larval-like morphologies into adulthood (Marliave and Peden 1989). Exploration of potential patterns of paedomorphy in Baikal sculpins should be a focus of future investigations into their skeletal evolution.

### Pelagic body shape: evolutionary parallelism

Beyond reduced skeletal density, we observe parallel shifts toward elongated body shapes in the pelagic sculpins (Fig. 5). Elongate morphologies reduce drag and enhance swimming efficiency, whereas deep-bodied benthic forms favor maneuverability within structured habitats but at the cost of swimming speed (Webb 1982, 1984; Hatfield and Schluter 1999). Similar transitions have evolved repeatedly across teleosts during benthic-to-pelagic shifts, in species both with and without swim bladders (Friedman et al. 2020a; Robinson and Wilson 1994; Walker 1997; Cooper et al. 2010; Rincon-Sandoval et al. 2020). The mechanistic basis of this morphological parallelism remains unresolved but likely reflects shifts toward a hydrodynamic optimum for swimming efficiency, as well as developmental and genetic constraints that could channel evolution along similar mutational trajectories during benthic to pelagic transitions. Notably, body elongation is also observed in *Leocottus*, the benthic sister group to *Cottocomephorus*, though to a lesser degree than in the pelagic species (St. John et al. 2022). Elongation is common in deep sea (Neat and Campbell 2013) and can also be advantageous in benthic habitats, especially those with complex structure, as seen in eels and other crevice-associated species (Friedman et al. 2020b). *Leocottus* further clusters with *Cottocomephorus* in morphospace (Sandel et al. 2025), suggesting similar morphologies may have arisen prior to the benthic-to-pelagic transition of *Cottocomephorus*.

We also find evidence for parallel evolution in fin placement between benthic and pelagic sculpins. While benthic sculpins possess lower, ventrally inserted pectoral fins used for station-holding on the substrate, pelagic species display pectoral fins inserted higher on the flanks. This higher, more horizontal orientation is thought to help the benthopelagic *Cottocomephorus* transition more readily from resting to forward propulsion (Taliev 1955). Although not quantified here, pectoral fins are also greatly elongated in *Comephorus* and *Cottocomephorus*, which increases surface area for stabilizing hydrodynamic drag and buoyancy (Jakubowski et al. 2003; Sideleva 2003). This functional trade-off parallels the silverspotted sculpin, *Blepsias cirrhosus*, where reduced ventral fin development and higher aspect ratios correlate with epi-benthic sculling rather than anchoring (Kane and Higham 2012). Thus, shifts in fin insertion likely reflect a dual pressure: the loss of station-holding utility and the increased demands of pelagic hydrodynamics.

Cottid fishes are almost entirely benthic with highly conserved morphologies (Buser et al. 2019, 2024). The divergence from benthic morphospace and reduction in skeletal density in pelagic Baikal sculpins highlights an ancestral capacity within cottids to diversify along the benthic-pelagic axis. This potential is likely constrained in their shallow freshwater relatives by a lack of ecological opportunity, specifically the shallow nature of most lakes and streams and lower prey diversity in boreal and temperate climates (Buser et al. 2024). Notably, only five of the ~40 Baikal species are pelagic or benthopelagic (Sandel et al. 2025), a pattern also consistent with a broader trend in teleost fishes in which benthic clades are typically more speciose and morphologically diverse than pelagic ones (Friedman et al. 2020a; Rincon-Sandoval et al. 2020).

### Pathways to pelagicism

The genetic and developmental mechanisms underlying these skeletal density reductions and body shape changes remain unresolved. Parallel evolution within Baikal sculpins, alongside broader patterns of convergence across Perciformes, including snailfishes (Gerringer et al. 2021) and notothenioids (Eastman et al. 2014), positions this clade of fishes as a powerful comparative system for understanding the genetic underpinnings of skeletal development and density regulation. The repeated evolution of similar skeletal phenotypes across pelagic perciform taxa provides a foundation for the use of comparative genomic tools to identify molecular pathways undergoing parallel evolutionary changes in tandem with convergently evolved traits (Hiller et al. 2012; Hilgers and Hiller 2025).

A potential cellular mechanism for skeletal density reduction in sculpins and notothenioids involves the shared developmental origin of osteoblasts and adipocytes. Both secondarily pelagic sculpins and notothenioids are exceptionally lipid-rich in multiple tissues. For example, *C. baikalensis* accumulates triglycerides during early growth, eventually reaching a remarkable ~39% lipid by wet weight (Kozlova and Khotimchenko 2000). Lipid accumulation also occurs in the neutrally buoyant notothenioids such as *Pleuragramma antarcticum*, which possesses specialized subcutaneous and intramuscular lipid sacs (Devries and Eastman 1978; Eastman 1988). Further, other deepwater marine fishes exhibit lipid-rich bone tissue (Lee et al. 1975), and, in mammals, osteoporosis is associated with elevated bone marrow adiposity (Muruganandan et al. 2018). Because osteoblasts and adipocytes arise from mesenchymal stem cells (Chen et al. 2016; Hu et al. 2018; Aghebati-Maleki et al. 2019; Pittenger et al. 2019), these observations suggest possible correlated trait evolution between skeletal density reduction and increased lipid storage via regulation of mesenchymal stem cell differentiation. Comparative genomic tools may be useful in unraveling the molecular mechanisms underlying these convergently evolving, correlated changes to lipid metabolism and skeletal density.

Benthic-to-pelagic transitions involve coordinated changes across tissue systems. A recent comparative transcriptomic analysis of *Comephorus* muscle revealed widespread gene expression changes, even between *Comephorus* species, including upregulation of the peroxisome proliferator-activated receptor (PPAR) pathway, a key regulator of adipogenesis, along with genes involved in sarcomere structure, calcium handling, and motor proteins, consistent with adaptations for sustained locomotion (Sapozhnikova et al. 2025). Extending similar analyses to other tissues will help clarify the molecular basis of these complex, multi-tissue transitions, particularly whether similar molecular mechanisms underlie parallel morphological and behavioral changes between *Comephorus* and *Cottocomephorus*.

### Summary

Here, we characterized patterns of skeletal density across the radiation of Baikal sculpins. We found that pelagic lineages have independently evolved reduced skeletal density, likely as a buoyancy mechanism. Pelagic fishes exhibited consistent declines in bone mineral density, contrasting with the more marginal and inconsistent shifts in bone thickness and porosity. These reductions in skeletal density were accompanied by convergent overall changes to body shape, including more streamlined morphologies and a shift in the position of the pectoral fins, mirroring a common teleost-wide trend in benthic-to-pelagic transitions. Future work should leverage these convergent phylogenetic patterns to dissect the molecular basis of skeletal density evolution and benthic-to-pelagic transitions.

## Supplementary Material

obag026_Supplemental_File Figs. S1 to S3, Tables S1 to S5, Supplementary Methods

## Data Availability

All code used for data processing and analyses is publicly available on GitHub (https://github.com/Brayan695/Parallel-reduction-in-skeletal-density-during-benthic-to-pelagic-transitions-in-Baikal-sculpins).
