# Supplementary material for: Parallel Reduction in Skeletal Density During Benthic to Pelagic Transitions in Baikal Sculpins
Source: Integr Org Biol. 2026 Jun 5;8(1):obag026. doi: 10.1093/iob/obag026 (PMC13383080; doi:10.1093/iob/obag026)
Supplement: obag026_Supplemental_File — Figs. S1 to S3, Tables S1 to S5, Supplementary Methods [file obag026_supplemental_file.docx]

**Supplemental Figures**


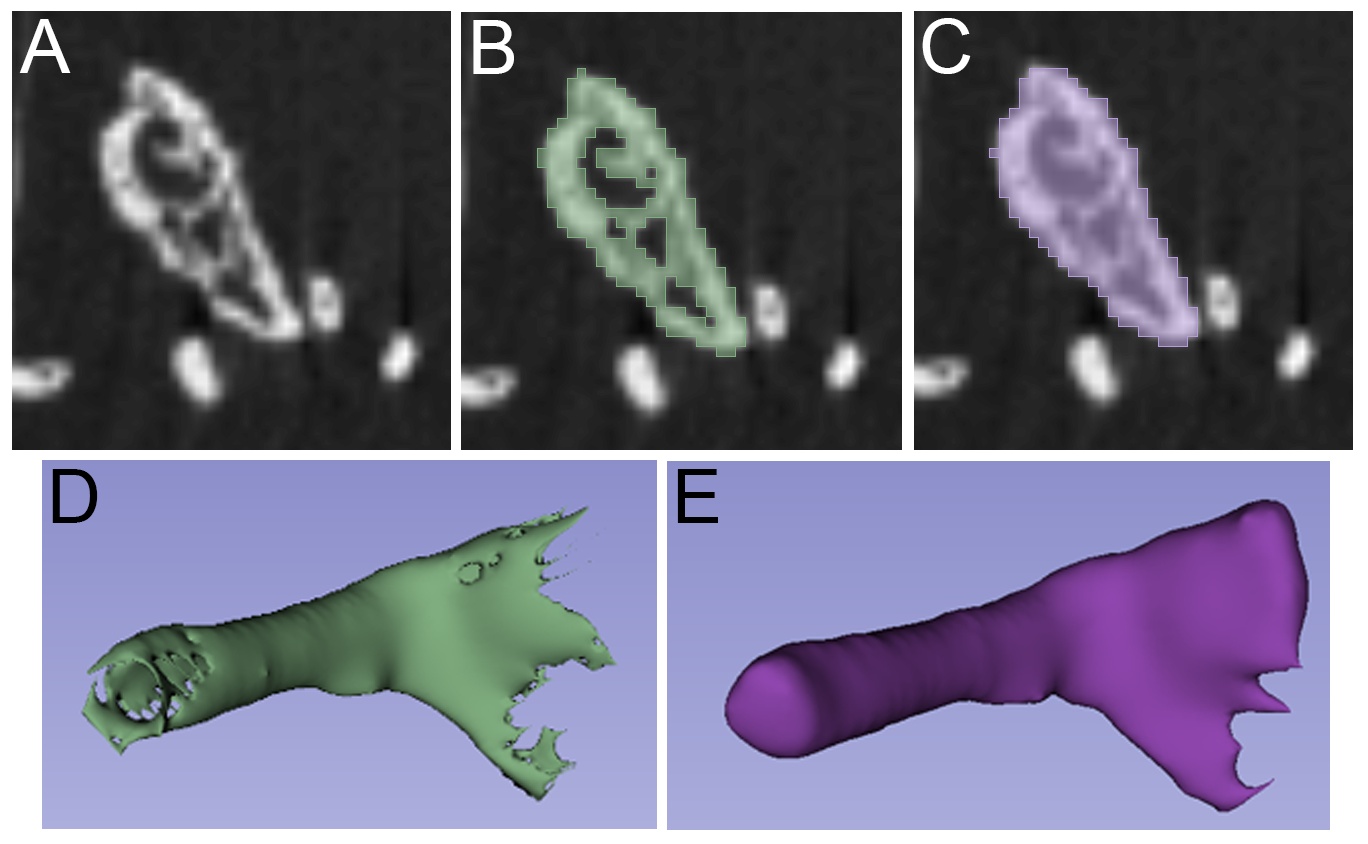


**Fig. S1. Methodology for estimating bone volume fraction (BV/TV)**. A) Representative CT cross-section of the ceratohyal. B) Segmentation of mineralized tissue used to calculate Bone Volume (BV). C) Total Volume (TV) defined by filling internal voids. D) 3D reconstruction of the segmented BV. E) 3D reconstruction of the TV.

#


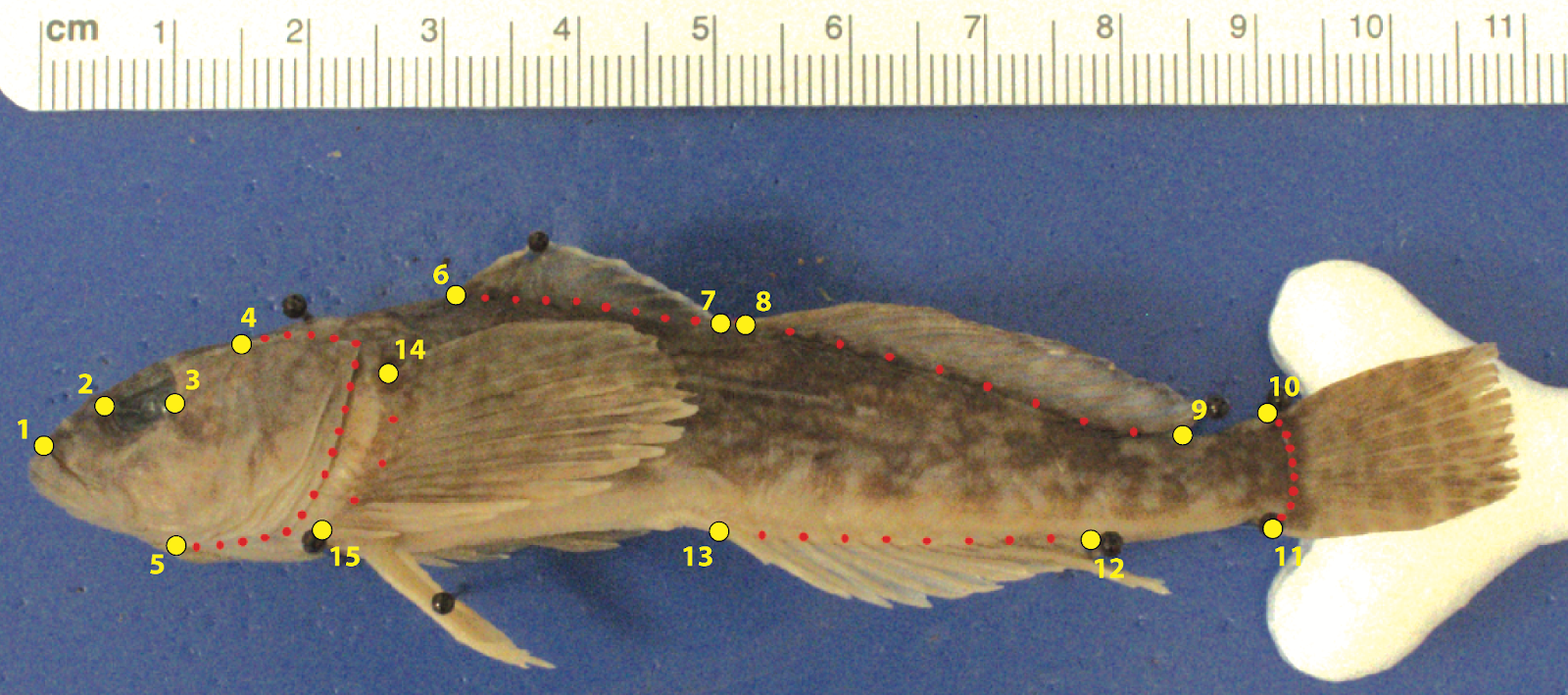


**Fig. S2. Anatomical landmarks for geometric morphometrics**. Example with *Paracottus knerii*. Primary landmarks (yellow): snout tip (1), orbital width (2-3), operculum (4-5), base of spinous dorsal fin (6-7), base of soft-rayed dorsal fin (8-9), base of caudal fin (10-11), base of anal fin rays (12-13), base of pectoral fin (14-15). The semi-landmarks are listed in red. There were 18 semi-landmarks for the operculum and 8 semi-landmarks each for the base of the spinous and soft-rayed dorsal fins, the caudal fin, and the anal fin. There were 3 semi-landmarks used for the base of the pectoral fin.

#

# **
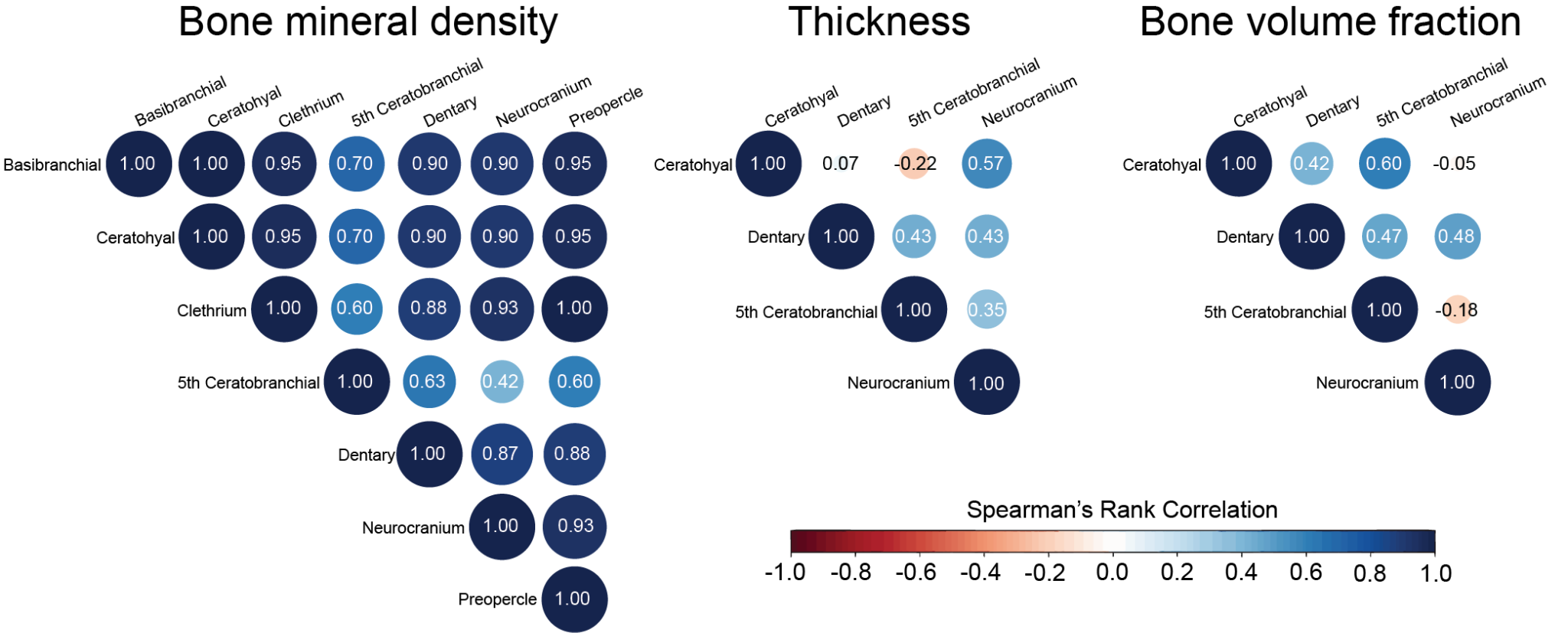
**

**Fig. S3. Spearman rank correlations among skeletal elements for density metrics**. Circle size and color indicate Spearman’s ρ, with values labeled for each comparison. To account for phylogenetic relationships, species mean values for each metric were transformed into phylogenetic independent contrasts.

# **Supplemental Tables**

**Table S1 -** Fish total length (TL) data for scanned specimens

| **Species** | **Sample**  **Avg TL (mm)** | **Species**  **Max TL (mm)†** | **Species**  **TL at Maturity (mm)**‡ |
| --- | --- | --- | --- |
| *Abyssocottus platycephalus* | 96 | 110 | 80-90 |
| *Abyssocottus pallidus* | 113 | 150 | 84-115 |
| *Abyssocottus thermalis* | 114 | 160 | n.d. |
| *Adipocottus multiradiatus* | 122 | 175 | n.d. |
| *Comephorus dybowski* | 120 | 130-140 | 75-95 |
| *Comephorus baikalensis* | 141 | 130-210 | 105-118 |
| *Cottocomephorus grewingkii* | 99 | 135-145 | 63 |
| *Cottocomephorus inermis* | 94 | 190-210 | 125 |
| *Paracottus knerii* | 92 | 80-140 | 61 |
| *Procottus jeittelesii* | 56 | 175 | 95 |

† maximum total length data aggregated from Bogdonovic 2023. Ranges reflect gender differences.

‡ length at maturity from Sideleva 2003 (A. platycephalus, A. pallidus, C. grewingkii, C. inermis, P. jeittelesii, and P. knerii), Jakubowski et al 2003 and Koryakov 1964 (C. baikalensis and C. dybowski). n.d. indicates no data.

## **Table S2** - List of anatomical landmarks and semi-landmarks used for geometric morphometrics

| **Anatomical Structure** | **Anchor Landmarks Number(s)** | **Number of Semi-Landmarks** |
| --- | --- | --- |
| Snout Tip | 1 | n/a |
| Orbital Width | 2,3 | n/a |
| Operculum | 4,5 | 18 |
| Base of spinous dorsal fin | 6,7 | 8 |
| Base of soft-rayed dorsal fin | 8,9 | 8 |
| Base of caudal fin | 10,11 | 8 |
| Base of anal fin Rays | 12,13 | 8 |
| Base of pectoral fin | 14,15 | 3 |

**Table S3** - Average phylogenetic ANOVA *P*-values for difference in bone properties between benthic and pelagic species. n.t. indicates not tested

| **Bone** | **Bone Mineral Density** | | **Bone Volume Fraction** | | **Thickness** | |
| --- | --- | --- | --- | --- | --- | --- |
|  | P | Hedges’ *g* | P | Hedges’ *g* | P | Hedges’ *g* |
| basibranchial | 0.0539 | -2.29 | n.t. | n.t. | n.t. | n.t. |
| ceratohyal | 0.0137 | -3.27 | 0.5130 | -0.73 | 0.0340 | -2.62 |
| cleithrum | 0.0845 | -1.99 | n.t. | n.t. | n.t. | n.t. |
| ceratobranchial | 0.0710 | -1.08 | 0.8455 | -0.22 | 0.5672 | -0.64 |
| dentary | 0.0047 | -4.19 | 0.1841 | -1.48 | 0.1334 | -1.69 |
| neurocranium | 0.0901 | -1.95 | 0.1633 | -1.56 | 0.0069 | -3.84 |
| preopercle | 0.0523 | -2.31 | n.t. | n.t. | n.t. | n.t. |

**Table S4** - Spearman rank correlation coefficients between the various bone density and centroid size.n.t. indicates not tested

| **Bone** | **Bone Mineral Density** | | **Bone Volume Fraction** | | **Thickness** | |
| --- | --- | --- | --- | --- | --- | --- |
|  | ρ | P | ρ | P | ρ | P |
| basibranchial | -0.50 | 0.17 | n.t. | n.t. | n.t. | n.t. |
| ceratohyal | -0.50 | 0.17 | -0.55 | 0.12 | 0.32 | 0.40 |
| cleithrum | -0.62 | 0.08 | n.t. | n.t. | n.t. | n.t. |
| ceratobranchial | -0.35 | 0.36 | -0.28 | 0.46 | 0.07 | 0.86 |
| dentary | -0.72 | 0.03 | -0.53 | 0.14 | 0.08 | 0.83 |
| neurocranium | -0.57 | 0.11 | -0.75 | 0.02 | -0.08 | 0.83 |
| preopercle | -0.62 | 0.08 | n.t. | n.t. | n.t. | n.t. |

**Table S5** - Spearman rank correlation coefficients between habitat depth and density. n.t. indicates not tested

| **Bone** | **Bone Mineral Density** | | **Bone Volume Fraction** | | **Thickness** | |
| --- | --- | --- | --- | --- | --- | --- |
|  | ρ | P | ρ | P | ρ | P |
| basibranchial | -0.25 | 0.52 | n.t. | n.t. | n.t. | n.t. |
| ceratohyal | -0.32 | 0.41 | -0.68 | 0.042 | -0.53 | 0.14 |
| cleithrum | -0.10 | 0.80 | n.t. | n.t. | n.t. | n.t. |
| ceratobranchial | -0.017 | 0.97 | 0.10 | 0.80 | 0.15 | 0.70 |
| dentary | -0.10 | 0.80 | -0.017 | 0.97 | 0.30 | 0.43 |
| neurocranium | -0.25 | 0.52 | -0.60 | 0.088 | -0.15 | 0.70 |
| preopercle | -0.17 | 0.67 | n.t. | n.t. | n.t. | n.t. |

# **Supplemental Methods**

## *Micro-CT Scan and Reconstruction Settings*

Specimens were scanned using a Bruker SkyScan 1273 micro-CT scanner configured with 70 kV X-ray voltage, 214 µA current, medium focal spot size, and 0.3° rotation step. Scan lengths ranged from 145 to 245 mm with voxel sizes between 12 and 20 µm, optimized for each specimen's size. Two calcium hydroxyapatite phantom markers (25% and 75% density) were included in each scan for bone mineral density calibration.

Micro-CT data reconstruction used Bruker NRecon v.2.1.0.1, cropping the region from the snout to the 6th or 7th vertebra. Reconstruction involved histogram adjustments with a zero lower bound and standardized upper bound. Data downsampling in DataViewer employed "Single Volume of Interest" with resize factors of 2 or 3, no color or intensity corrections were performed during downsampling.. Amira segmentation created LabelFields using paintbrush tools with interpolation (Ctrl+I), while phantoms were segmented separately using masking functions and magic wand tools with consistent pixel brightness thresholds. Ring artifact reduction set to six for improved image quality. For oversized specimens, we performed multiple overlapping scans stitched into composite datasets. Large files were reduced using Bruker DataViewer v.1.5.4 with downsampling factors of 2 or 3. This standardized protocol with phantoms in every scan ensured consistency across specimens and species, making bone property data directly comparable among Baikal sculpins in the present dataset.

## *Computation of Bone Mineral Density*

Material statistics were extracted from Amira’s measurement tool, which provided voxel count and cumulative brightness sum for each segmented region. Mean pixel brightness (MPB) was then computed separately as:

$MPB = \frac{Cumulative Sum}{Voxel Count}$ (1)

To establish a reference model, the MPB values of the 25% and 75% hydroxyapatite phantoms were used to create a linear regression model, correlating MPB with known density percentages. The general form of this model was:

$\%HA = m\times MPB+b$ (2)

where m represents the slope of the calibration curve derived from the phantom MPB values and their known hydroxyapatite percentages, and b is the intercept.

Once the calibration model was established, the MPB of each segmented bone was calculated from the material statistics and substituted into the model to determine its hydroxyapatite percentage:

${\%HA}_{bone} = m\times{{MPB}_{bone}}+b$ (3)

This method ensures that bone mineral density quantification remains standardized across different scans and specimens.

## *Standardized Density Visualization*

To standardize visualization across scans, colormap thresholds were adjusted to represent values from 10% to 90% hydroxyapatite:

$Min = \left( \frac{{MPB}_{75}-{MPB}_{25}}{50} \right)\times10+\frac{{MPB}_{25}}{\left( \frac{{MPB}_{75}-{MPB}_{25}}{50} \right)}$ (4)

$Max = \left( \frac{{MPB}_{75}-{MPB}_{25}}{50} \right)\times90+\frac{{MPB}_{25}}{\left( \frac{{MPB}_{75}-{MPB}_{25}}{50} \right)}$ (5)

These thresholds ensured that pixel intensities outside the target range were rendered as black (low) or white (high) for visual contrast.

## *Image Stack Loading and Visualization*

We imported reconstructed scan data consisting of log files and PNG slices into 3D Slicer using the Image Stacks module. The pixel size extracted from log files was converted from micrometers to millimeters and entered into the Spacing field in the Volumes Module under Volume Information. By adjusting slice skip parameters, we maintained total output sizes below 200 MB to ensure computational efficiency. For optimal bone visualization, we enabled volume rendering using the CT AAA preset while making adjustments to opacity mapping and region of interest boundaries.

## *Segmentation and Isolation of Bone Regions*

Using the Segment Editor module, we performed segmentation by first applying consistent thresholding across specimens (min: 30, max: 255) to standardize pixel intensity selection. We then refined the segmentations using paint, erase, and scissors tools to isolate bones of interest from surrounding structures. These segmented regions represented the bone volume (BV) used in subsequent bone volume fraction calculations.

## *Generation of Total Volume (TV)*

We created solid bone representations by cloning each isolated segmentation in the Data module and applying the Wrap Solidify tool to generate watertight total volumes (TV). This process filled both internal and external voids. When necessary, we performed additional manual segmentation refinements using paint, erase, and fill between slices tools.

## *Bone Volume Fraction Calculation*

We calculated bone volume fraction (BVF) as the ratio of bone volume (BV) to total volume (TV). Volume measurements were obtained through the Segment Statistics module, extracting BV and TV values using Label Map Statistics, Scalar Volume Statistics, or Closed Surface Statistics. Valid BVF values ranged between 0 and 1, with values exceeding 1 indicating segmentation errors that required corrections to the total volume segmentation.

## *Bone Thickness Measurement*

For thickness measurements, we exported BV segmentations as both Labelmap and Model files. Using the Simple Filters module, we processed these files through a binary thinning algorithm (BinaryThinningImageFilter) followed by a distance transformation (DanielssonDistanceMapImageFilter) to compute thickness at each model point. The Probe Volume With Model module then generated 3D thickness heatmaps where yellow indicated thicker regions and dark blue represented thinner areas.

## *Computation of Mean Bone Thickness*

We computed mean bone thickness using Python scripting within 3D Slicer. The script processed segmented bone models by extracting thickness values from thousands of surface points. For each model, the script retrieved thickness values stored in the point data and calculated the mean by averaging all measurements. This semi-automated approach ensured consistent specimen comparisons and enabled efficient quantification of mean bone thickness and porosity. The resulting standardized metrics revealed structural patterns that complemented our bone mineral density assessments.

##

## *Standard Visualization*

Micro-CT scan images of our Baikal sculpins (Figure 7) were processed in Amira using colormap settings calibrated to the minimum (4) and maximum (5) values established in Section 2.5.5. These threshold values, representing the 10% to 90% hydroxyapatite range, were selected to exclude potential measurement uncertainties at the extremes of our data range.

# 
